# Association between low bone mass and calcium and caffeine intake among perimenopausal women in Southern Brazil: cross-sectional study

**DOI:** 10.1590/1516-3180.2013.1315428

**Published:** 2013-10-01

**Authors:** Daniele Lazzarotto Harter, Fernanda Michielin Busnello, Raquel Papandreus Dibi, Airton Tetelbom Stein, Sérgio Kakuta Kato, Carla Maria De Martini Vanin

**Affiliations:** I BSc. Nutritionist, Department of Nutrition, Universidade Federal de Ciências da Saúde de Porto Alegre (UFCSPA), Porto Alegre, Rio Grande do Sul, Brazil.; II PhD. Adjunct Professor, Department of Nutrition, Universidade Federal de Ciências da Saúde de Porto Alegre (UFCSPA), Porto Alegre, Rio Grande do Sul, Brazil.; III MSc. Preceptor of Medical Residency Program in Gynecology, Santa Casa de Misericórdia de Porto Alegre, Porto Alegre, Rio Grande do Sul, Brazil.; IV PhD. Titular Professor, Department of Public Health, Universidade Federal de Ciências da Saúde de Porto Alegre (UFCSPA), Porto Alegre, Rio Grande do Sul, Brazil.; V MSc. Assistant Professor, Department of Public Health, Universidade Federal de Ciências da Saúde de Porto Alegre (UFCSPA).; Department of Statistics, Pontifícia Universidade Católica do Rio Grande do Sul (PUCRS), Porto Alegre, Rio Grande do Sul, Brazil.; VI PhD. Associate Professor, Department of Gynecology and Obstetrics, Universidade Federal de Ciências da Saúde de Porto Alegre (UFCSPA), Porto Alegre, Rio Grande do Sul, Brazil.

**Keywords:** Osteoporosis, Calcium, dietary, Caffeine, Ultrasonography, Body mass index, Osteoporose, Cálcio na dieta, Cafeína, Ultrassonografia, Índice de massa corporal

## Abstract

**CONTEXT AND OBJECTIVE:**

Osteoporosis is a skeletal abnormality characterized by reduction and alteration of bone microarchitecture that results in increased fragility and greater predisposition to fractures. Age and low bone mass are the main non-modifiable risk factors for osteoporotic fractures. The modifiable factors include sedentary lifestyle, inadequate calcium intake, excessive alcohol and/or caffeine consumption, smoking and low body weight. The aim here was to evaluate the association between low bone mass and calcium and caffeine intake among perimenopausal women in Southern Brazil.

**DESIGN AND SETTING:**

Cross-sectional study conducted in Porto Alegre and Canoas, Rio Grande do Sul, Brazil.

**METHODS:**

Women (n = 155) of mean age 53.6 ± 9.5 years were evaluated through a cross-sectional study in Southern Brazil. Food frequency questionnaires, bone mass evaluation using calcaneal ultrasound and anthropometric assessment were used.

**RESULTS:**

The prevalence of overweight was 67.7%. In the bone mass screening, 30.3% had low bone mass and 4.5% had osteoporosis. The median calcium intake was 574.94 mg/day and the caffeine intake was 108.11 mg/day. No association was found between bone mass and anthropometric parameters, calcium intake or caffeine intake. It was found that 38.4% of the women had low bone mass.

**CONCLUSIONS:**

No association was found between calcium and caffeine intake and bone mass. High prevalence of low bone mass was observed.

## Abstract (pt)

**CONTEXTO E OBJETIVO:**

A osteoporose é uma alteração esquelética caracterizada pela redução e pela alteração da microarquitetura óssea, que resultam em aumento da fragilidade e maior predisposição a fraturas. A idade e a baixa massa óssea são os principais fatores de risco não modificáveis para fraturas osteoporóticas. Os fatores modificáveis incluem sedentarismo, inadequada ingestão de cálcio, excessivo consumo de álcool e/ou cafeína, tabagismo e baixo peso corporal. O objetivo foi avaliar associação entre baixa massa óssea e ingestão de cálcio e cafeína por mulheres climatéricas no Sul do Brasil.

**TIPO DE ESTUDO E LOCAL:**

Estudo transversal realizado em Porto Alegre e Canoas, Rio Grande do Sul, Brasil.

**MÉTODOS:**

Mulheres (n = 155) com idade média de 53,6 ± 9,5 anos foram avaliadas em um estudo transversal na região Sul do Brasil. Foram utilizados questionários de frequência alimentar, avaliação da massa óssea por ultrassonometria de calcâneo e avaliação antropométrica.

**RESULTADOS:**

A preval ência de sobrepeso foi de 67,7%. No rastreamento de massa óssea, 30,3% apresentou baixa massa óssea e 4,5%, osteoporose. A ingestão mediana de cálcio foi de 574,94 mg/dia e de cafeína foi de 108,11 mg/dia. Não foi encontrada associação entre massa óssea e os parâmetros antropométricos, ingestão de cálcio ou de cafeína. Verificou-se que 38,4% das mulheres apresentaram perda de massa óssea.

**CONCLUSÕES:**

Não foi encontrada associação entre a ingestão de cálcio e cafeína com a massa óssea. Foi observada alta prevalência de baixa massa óssea.

## INTRODUCTION

Osteoporosis is a metabolic disease characterized by reduced bone mass, fragility of bone microarchitecture and consequent reduction in bone resistance, resulting in greater susceptibility to fractures.^1,^ ^2^ These occur with higher frequency in places where there is a greater proportion of trabecular bone, since this type of bone has a higher rate of remodeling than shown by cortical bone, and it is more susceptible to estrogen deficiency.^2,3^ Increasing prevalence of osteoporosis is projected around the world as a result of growing life expectancy and the consequent increase in the elderly population. It is the most common metabolic disease and the main cause of fractures due to skeletal fragility, and is considered to be one of the main public health problems because of the individual and social repercussions.^4,5^

Age and low body mass are the main risk factors for osteoporotic fractures. The risk factors that contribute towards reduced bone mass can be classified either as non-modifiable factors, such as hereditariness, ethnicity (Caucasian), age, female sex and the individual's hormonal situation; or as modifiable or environmental factors, which include sedentary lifestyle, inadequate calcium intake, excessive consumption of alcohol and/or caffeine, smoking and low body weight.^6^

The daily intake recommendations in effect for nutrients, according to the dietary references intakes (DRIs) from the National Academy of Sciences (2006) are 1000 mg/day of calcium for women from 31 to 50 years of age and 1200 mg/day for women over 50 years.^7^ Dairy products, such as milk, cheese, yogurt and other milk by products, are frequently used for preventing bone loss because, besides being richer sources of calcium, they are also sources of phosphorus, magnesium, potassium, zinc and protein, and they are present in typical diets.^8^ Appropriate intake of calcium and vitamin D is important for bone health, and is recognized as an important component in the drug prescription regime for osteoporosis.^9^ Several studies conducted over recent years have correlated caffeine intake with calcium absorption. Most of them showed that moderate consumption of this substance does not harm bone health.^10-12^ However, excessive doses of caffeine may result in higher calcium excretion, thus increasing the risk of osteoporosis.^13,14^ The effects of caffeine on bone tissue have been correlated with increased calciuria and decreased efficiency of intestinal absorption of calcium.^15^ These mechanisms may promote a negative balance of calcium metabolism, thus presenting a negative impact on bone metabolism and leading to significantly greater bone mass reduction. However, a balanced diet, with the proper level of calcium intake and coffee consumption limited to three cups (equivalent to 710 ml) per day (300 mg/day of caffeine), may reduce the risk of osteoporosis and fractures, especially in the elderly population.^16^

Because of the importance of nutritional factors (especially calcium and caffeine) on bone mass, this study evaluated the calcium and caffeine intake among menopausal women and its relationship with bone mass. 

### OBJECTIVE

To evaluate the association between bone mass and calcium and caffeine intake among perimenopausal women in Southern Brazil. 

### METHODS

This was a cross-sectional study with data collected between March and May 2010, using questionnaires in the cities of Porto Alegre and Canoas, Rio Grande do Sul, Brazil. This study formed part of a bigger study aimed at identifying the risk factors for osteoporosis and low bone mass, which include: physical exercise, smoking, alcohol use, use of corticosteroids and diet (calcium, caffeine and vitamin D intake).

### Study sample

The estimated sample size for screening low bone mass using calcaneal ultrasound was 157 subjects, sampled consecutively,^17^taking into consideration a confidence level of 95%, error of 6% and an approximate prevalence of medium and high risk of low bone mass of 18%. 

Women 40 years of age or older were included. Pregnant and illiterate women were excluded. Pregnant subjects were excluded because of the different pattern of their anthropometric parameters. The questionnaires were self-administered, and therefore illiterate women were not eligible. The subjects were selected at the Air Force Hospital of Canoas and at the Municipal Cleansing Department of Porto Alegre. 

### Menopausal status

Menopausal status is a retrospective diagnosis that is a landmark of the climacteric period. It corresponds to the last menstrual cycle, which is only recognized as such after 12 months of amenorrhea, and is usually reached around the age of 50.18 This information was based on self-reported questionnaires.

### Nutritional profile 

An evaluation of the nutritional status was conducted based on measurements of weight (kg), height (m), abdominal circumference (AC, in cm) and tricipital skinfold (TSF, in mm). The body mass index (BMI, in kg/m^2^ ) and arm muscle circumference (AMC, in cm) were calculated. From the BMI, the women were classified as 1) low weight (presenting malnutrition), 2) eutrophic or 3) overweight or obese.

*Weight, height and body mass index (BMI)*

A digital platform scale with capacity of up to 150 kg was used to determine the subjects' weight. Individuals were weighed barefoot and only wearing light clothes.^19^

Height was measured using the anthropometric measuring stick of the digital platform scale. For this measurement, the individuals were barefoot, with their weight equally distributed between their feet, arms extended down the sides of their bodies and heels together, touching the vertical rod of the measuring stick, and with their head straight and eyes looking forward.^19^

The body mass index (BMI) was calculated using the formula BMI = weight (kg)/height2 (m). The most widely used BMI classification follows the proposal from the World Health Organization (WHO) committee (1995/1998),^20,21^ and this was used to classify women under the age of 60. For women aged 60 years or over, BMI was classified in accordance with Lipschitz,^22^ because this proposal takes into account the changes in body composition that occur with aging. Thus, the classifications in Table 1 were used.

**Table 1:** Body mass index (BMI) classification for adults20,21 and elderly

Anthropometric index	Cutoff points	Classification of nutritional status
BMI (WHO)	≤ 18.5 kg/m2	Low weight
	18.5 - 24.9 kg/m2	Eutrophic
	≥ 25.0 kg/m2	Overweight
BMI (Lipschitz) for women 60 years old or over	< 22.0 kg/m2	Low weight
	22.0 - 27 kg/m2	Eutrophic
	> 27.0 kg/m2	Overweight

*Abdominal circumference (AC)*

Abdominal circumference (AC) was measured using a nonextendable flexible tape made of a resistant material, with a precision of 0.1 cm. The measurement was made at the end of expiration, around the abdominal region at the midpoint between the lower costal margin and the iliac crest, seen from the front, as defined and recommended by WHO, 1995.^20^ The AC was classified in accordance with the WHO reference:^21^ low risk of metabolic complications, with abdominal circumference up to 80 cm; increased risk of metabolic complications, with abdominal circumference ≥ 80 cm; and substantially increased risk of metabolic complications, with abdominal circumference ≥ 88 cm.

*Tricipital skinfold (TSF)*

The tricipital skinfold (TSF) was measured vertically at the midpoint of the arm, between the acromion and the olecranon at the posterior face of the non-dominant arm, using a scientific plicometer with sensitivity of 0.1 mm and reading range of 78 mm. Three measurements were made and their arithmetic mean was used.^19^ The results were classified in accordance with the reference parameters suggested by Blackburn et al.,^23^ and the formula used was: TSF adjustment (%) = TSF (mm)/TSF 50^th^ percentile x 100. The 50^th^percentile values for TSF, AC and AMC followed the Frisancho reference^24^ for women up to 74.9 years old and the NHANES III reference for women over 75 years of age.^25^ Taking into consideration the percentage adjustments, individuals were classified as presenting serious malnutrition (< 70%), moderate malnutrition (70-80%), slight malnutrition (80-90%), eutrophia (90-110%), overweight (110-120%) or obesity (> 120%).

*Mid-arm circumference (MAC)*

The mid-arm circumference (MAC) was measured at the midpoint between the acromion and the olecranon using a measuring tape. The results were classified in accordance with the reference parameters suggested by Blackburn et al.,^23^ and the formula used was: adjusted MAC (%) = MAC (cm)/MAC 50^th^ percentile x 100. Again, taking into consideration the percentage adjustments, individuals were classified as presenting serious malnutrition (< 70%), moderate malnutrition (70-80%) slight malnutrition (80-90%), eutrophia (90-110%), overweight (110-120%) or obesity (> 120%).

*Arm muscle circumference (AMC)*

To obtain the arm muscle circumference (AMC), the MAC and TSF values were used in the following formula: AMC (cm) = MAC (cm) - [TSF (mm) x 0.314]. The results were classified in accordance with the references suggested by Blackburn et al.,^23^ and the formula used was: adjusted AMC (%) = AMC (cm)/AMC 50^th^ percentile x 100. Classifying the individuals according to the adjustment percentages gave the following: serious malnutrition (< 70%), moderate malnutrition (70-80%), slight malnutrition (80-90%) and eutrophia (> 90%). 

### Calcium and caffeine

To quantify the caffeine and calcium intake in the diet, two instruments were constructed following the model of the validated semi-quantitative food frequency questionnaire (FFQ) proposed by Heath et al.,^26^ with adaptations for the population studied. The FFQ estimates the individual's habitual intake and offers the possibility of stratifying the results into consumption categories. This is considered to be the most practical and valuable method for evaluating food intake, and it is of utmost importance in epidemiological studies that correlate the diet with occurrences of chronic non-transmissible disease.^27^ These instruments are selfapplicable, which justifies exclusion of illiterate subjects. 

The consumption of foods that are sources of and/or rich in calcium, like dairy products, soya milk, tofu, eggs, fish, oleaginous fruits, dark green vegetables, cooked beans and black bread, was quantified in household measurements and then transformed into grams. The same was done to determine the caffeine intake, through consumption of roasted and ground coffee, instant coffee, black tea, hot chocolate, chocolate in bars, soft drinks and chimarrão (yerba mate tea). The following responses regarding consumption frequency were used: never, less than once a month, one to three times per month, once a week, two to four times per week, once a day and two or more times per day, following the model suggested by Fisberg et al.^27^ The quantities considered for calculating nutrient intakes were obtained from food composition tables,^28-31^ and from the nutritional information on food packages when these foods were not included in the tables. Calcium and caffeine intakes (mg/day) were described in terms of the median and interquartile range.

### Low bone mass (LBM) screening

Low bone mass (LBM) screening was conducted using calcaneal quantitative ultrasound (QUS) because of its advantages: low cost, convenience, short examination time and lack of ionizing radiation.^32^ The bone mass was determined using a portable ultrasound device (SONOST-2000, OsteoSys Co. Ltd.), and the results were used as a predictor for fractures.^33-35^ The reasons for choosing the calcaneus bone were that it has a high proportion of trabecular bone in its structure, a flat and parallel shape for the lateral and medial surfaces and, since this is a weight-bearing bone, it can simulate the properties of the proximal femur. Bone mineral density (BMD) from densitometry and QUS were used as predictors for any type of fracture and for osteoporotic fractures, regardless of BMD.^36^ In the same way as for BMD, the results were classified as losses of standard deviations (SD) in relation to normal young adult controls.^37^ Thus, QUS can be classified as: 1- minimal fracture risk with a loss of up to 1 SD below peak bone mass; 2- increased fracture risk with a loss of up to 1 to 3 SD below peak bone mass; 3- high fracture risk with a loss of over 3 SD below peak bone mass.

### Statistical analysis 

The qualitative variables were described through absolute and relative frequencies and quantitative variables through means and standard deviations or medians and interquartile ranges. In order to compare the means of anthropometric measurements in relation to bone mass classification, the Student t test was used. Comparison of calcium and caffeine consumptions in relation to bone mass classification was done using the non-parametric Mann-Whitney test. In all analyses, the significance level of 5% (P ≤ 0.05) was accepted. The database was constructed and the statistical analyses were conducted using Statistical Package for Social Sciences (SPSS) version 17.0.

### Ethical considerations

This study was approved by the Research Ethics Committee of Irmandade Santa Casa de Misericórdia of Porto Alegre (CEP/ISCMPA), under no. 257/09. The ethical principles followed were in accordance with the guidelines from Resolution 196/96 of the National Health Council. Participation in the study among the women evaluated was dependent on their signing an informed consent statement.

### RESULTS

Out of 217 eligible subjects, three declined to give responses in the survey and 59 did not have complete anthropometric and/or calcaneal ultrasound measurements. Therefore, 155 women were enrolled in the study. Their mean age was 53.6 ± 9.5 years, ranging from 40 to 84 years. Among the subjects, 60.6% (n = 94) were postmenopausal and 39.4% (n = 61) were premenopausal, according to the self-reports. In screening using calcaneal ultrasound, it was found that 65.2% of the women (n = 101) presented a normal bone mass pattern; 30.3% (n = 47), low bone mass; and 4.5% (n = 7), osteoporosis. 

**Figure 1:**
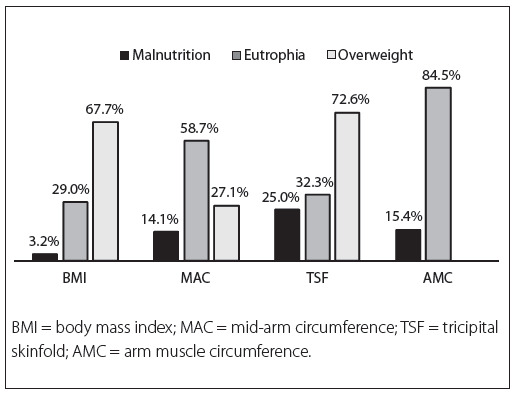
Percentage distribution of different nutritional statuses according to different methods of anthropometric evaluation in a population of perimenopausal women in Southern Brazil, 2010.

With regard to nutritional profile, the frequencies of different nutritional statuses according to BMI and the adjusted percentages for MAC, TSF and AMC are demonstrated in Figure 1. A large AC (greater than or equal to 80 cm) was observed in 27% (n = 42), while 60% (n = 93) had a very large AC (greater than or equal to 88 cm).

There were no significant differences in the average BMI, AC, MAC, TSF and AMC measurements, in relation to low bone mass, using the Student t test (Table 2).

**Table 2:** Association between anthropometric parameters and bone mass classification using calcaneal ultrasound in a population of perimenopausal women in Southern Brazil, 2010; means and standard deviations

	Bone mass classification				P-value
Variables	Normal		Low		
	Mean	SD	Mean	SD	
BMI (kg/m2)	27.86	4.61	27.78	5.23	0.905
AC (cm)	91.26	10.34	91.27	11.76	0.995
MAC (% adjustment)	103.52	16.38	101.21	13.96	0.381
TSF (% adjustment)	108.84	28.62	106.61	30.25	0.652
AMC (% adjustment)	100.15	12.46	99.27	11.09	0.665

### Calcium and caffeine

There was high variability in calcium and caffeine consumption among individuals. The median calcium consumption was 548 mg/day (range: 262-870) and median caffeine consumption was 108 mg/day (range: 60-170). Low bone mass and osteoporosis were grouped for the statistical analyses because of the low frequency of osteoporosis in the sample. Using the nonparametric Mann-Whitney test, no statistically significant difference in calcium (P = 0.86) or caffeine consumption (P = 0.69) was found in relation to the presence of low bone mass (Table 3).

**Table 3:** Association between bone mass classification using calcaneal ultrasound and calcium and caffeine intake in a population of perimenopausal women in Southern Brazil, 2010; median and interquartile range (IQR)

	Bone mass classification				P-value
Variables	Normal		Low		
	Median	IQR	Median	IQR	
Calcium (mg/day)	535.98	261.93 - 862.21	604.55	279.71 - 889.71	0.860
Caffeine (mg/day)	108.00	64.13 - 179.15	107.50	55.71 - 162.00	0.694

### DISCUSSION

The prevalence of overweight of 67.7% shown by the BMI in this study was similar to what was found among Brazilian women according to the Consumer Expenditure Survey (CES) data of 2009 (65%).^38^ However, the CES used the WHO classification (overweight starting at BMI of 25 kg/m^2)^ for all adult individuals, regardless of age. Therefore, individuals with BMI > 27 kg/m^2^ and age greater than or equal to 60 years, were classified as overweight. Thus, a higher tendency towards overweight was observed in this study, taking into consideration the different cutoff points used in the BMI evaluation. The prevalence of overweight was similar to the CES data from Southern Brazil, where the prevalence was 71.2%. The prevalences of different nutritional statuses differed between the methods evaluated, but the prevalence of overweight was still high. It should be noted that in the AMC classification, there is no standard for overweight, since this measurement evaluates muscle reserves, excluding lipid reserves,^19^ and therefore, some individuals considered overweight using other methods were classified as eutrophic according to the AMC. 

A few studies have been conducted on similar populations using the same classification criteria, in order to screen for low bone mass using QUS. Considering that the methodology used for screening of low bone mass in the present study was the same as applied by Steiner et al.,^39^ it was possible to make a comparison between the findings. In our findings, the prevalence of low bone mass was three times greater and the prevalence of osteoporosis was two times greater than what had been found by these authors. The presence of osteoporosis in our study was lower than what was found in a study conducted among African-American women of mean age 54 ± 7 years, in which bone mass was determined by means of QUS using the same criteria as in the present study. In that study, it was found 23.3% of the sample had osteopenia and 9.3% had osteoporosis.^40^

Despite the advantages of using bone ultrasound, there are some controversial issues regarding the technique, in relation to the precision of and result from coefficients of variation. However, it is a good method when the main objective is to screen for LBM, which is a good predictor of fractures.^34^

The anthropometric variables did not show any association with bone mass in our study, but Parisi Júnior et al. evaluated 314 women with an average age of 60.2 ± 9.96 years and observed that those with a mean BMI of 25.82 ± 4.33 kg/ had low bone mass, those with a mean BMI of 25.34 ± 3.85 kg/m^2^ had osteoporosis and those with a mean BMI of 27.02 ± 4.98 kg/m^2^ had normal bone mass, although bone mass was evaluated by means of bone mineral densitometry using dual energy x-ray absorptiometer (DEXA).^41^ In the study conducted by Steiner et al., in which the risk of osteoporosis among 461 women of mean age of 60 ± 9.0 was evaluated by means of calcaneal ultrasound, a positive relationship was observed between bone mass and BMI (P = 0.006): women with BMI of 29.1 ± 4.9 kg/m^2^ presented low risk of osteoporosis; those with BMI of 27.3 ± 4.5 kg/m^2^ presented an average risk and women with a BMI of 26.9 ± 6.0 kg/m^2^ presented high risk.^39^ Thus, these studies suggest that higher BMI may provide a protective effect, regardless of the methodology used for evaluating bone mass.

In a randomized, double blind, placebo-controlled study conducted by Palacios et al., the means and standard deviations for calcium consumption among adults in the control group were 668 ± 273 mg/day according to food intake recall, and 463 ± 325 mg/day according to a food frequency questionnaire.^42^ In the present study, an average consumption of 547.94 mg/day was found, which is a value similar to what was found by Palacios et al., taking into consideration the standard deviation.^42^ However, the values found in both studies were lower than the National Academy of Sciences references, i.e. the dietary references intake (DRI), in which an intake of 1000 mg/day for women aged 31 to 50 years and 1200 mg/day for women over 50 years is recommended.^7^ Caffeine intake also presented a result lower than the findings of Boggs et al.^43^ in a prospective cohort study in which the median intake was 312 mg/day, while in our findings, this intake was 108.11 mg/day. In a study conducted in Brazil among both white and black men, higher calcium intake was found in whites (720 ±346 versus 558 ± 236 mg), but there was only a correlation with the bone mineral density of the femoral neck among blacks.^44^ No other recent studies in Brazil evaluating caffeine intake in a sample similar to the present study were found. 

Like in other studies that used self-applicable questionnaires, it is believed that the consumption of foods that are sources of caffeine and calcium was underestimated in the present study due to large numbers of errors in filling out the instrument used for evaluating the diet intake of these nutrients. Although one of the advantages in using food frequency questionnaires is the possibility of self-administration, this method of evaluation requires correct explanation of the procedure by the evaluator and understanding of the procedure by the individual under evaluation. Moreover, there are limitations regarding external validity in using self-applicable questionnaires. Therefore, we believe that the values presented here for calcium and caffeine consumption may not truthfully represent the actual intake of the population. Individual application through the interviewer may reduce the errors in filling out the questionnaire, thus making the data more reliable, with greater accuracy, and reducing the variations between individuals. 

The results found in the present study with regard to associations with calcium and caffeine intake differ from those of Hallström et al., who observed that high coffee consumption (four or more cups per day), among both men and women, plus high calcium intake (more than 1200 mg/day), did not alter BMD compared with those who had high consumption of coffee and low (< 600 mg/day) or intermediate intake (600-1200 mg/day) of calcium.^45^ There is still no strong evidence to show that calcium alone has the capacity to reduce the risk of fractures.^9^ In a double-blind, placebo-controlled study that evaluated 830 postmenopausal women with an average age of 75 years, a significantly lower fracture rate was observed among the subjects who used calcium supplements.^46^ Although the menopause forms a landmark in relation to bone mass reduction because of its association with declining estrogen production caused by the loss of ovary function, aging itself presents a direct relationship with the risk factors and the increased risk of fractures. Therefore, older women (75 years of age or over) seem to obtain better benefits from dietary interventions and their impact on bone metabolism than seen among women who have been postmenopausal for shorter times.^9^

Most cross-sectional studies have not found any association between caffeine intake and changes in BMD.^12^ This study design is observational, and the subjects are observed on just one occasion. It is thus very difficult to infer a cause-effect relationship from such a study design. One common problem with this type of study is reverse causality, in which the outcome has caused the predictor. 

There are several different risk factors for osteoporosis, although the impact of caffeine consumption on bone metabolism is still unclear.^16^ Considering the physiopathology of this disease, there is a need to increase calcium consumption. In each life cycle, there are individual needs for calcium, in order to achieve an ideal bone mass. As the life span and number of chronic diseases increase, the importance of a balanced diet that is rich in calcium becomes reinforced. Thus, further studies are necessary in order to obtain more information that would allow guidance to be directed towards the population that is susceptible to low bone mass and osteoporosis.

Several limitations of the present study need to be pointed out, such as misclassification due to use of self-application questionnaires. On the other hand, the data collection technique was designed to avoid this limitation. A cross-sectional study collecting data with regard to current consumption of calcium and caffeine does not reflect the real intake of these nutrients during the peak phase of bone mass. 

### CONCLUSION

High prevalence of low bone mass was observed in the sample studied. The findings of this study did not show any statistically significant difference in calcium and caffeine intake with regard to bone mass. There is a need to conduct further studies in order to better understand the role of caffeine and its effect on bone metabolism in relation to osteoporosis. In order to help investigate the association of diet with bone mass, additional studies are suggested.
